# Nutritional Biochemistry

**DOI:** 10.3390/ijms24119661

**Published:** 2023-06-02

**Authors:** Shih Min Hsia

**Affiliations:** 1 School of Nutrition and Health Sciences, College of Nutrition, Taipei Medical University, Taipei 11031, Taiwan; 2 Graduate Institute of Metabolism and Obesity Sciences, College of Nutrition, Taipei Medical University, Taipei 11031, Taiwan; 3 School of Food and Safety, Taipei Medical University, Taipei 11031, Taiwan; 4 Nutrition Research Center, Taipei Medical University Hospital, Taipei 11031, Taiwan

Nutritional biochemistry involves a wide range of fields, and many studies on nutritional biochemistry have focused not only on uncovering the relationship between diet and disease but also on revealing the importance of nutritional intake in the development of cancer. To provide more basic research for clinical applications, a Special Issue of the *International Journal of Molecular Science* entitled “Nutritional Biochemistry” has collected five contributions of original articles that provide new insights into the use of active compounds to improve specific diseases or cancers. 

First, as mentioned in the study of Zhang et al. [1], many bioactive ingredients that regulate lipid metabolism can exert their effects through circadian clock-related mechanisms. It has been reported that piperine (1-piperonylpiperidine, PIP), the main irritating alkaloid component of black pepper (black pepper), has the effect of improving lipid metabolism both in vitro and in vivo; however, whether the circadian clock regulator is involved in the protective effect of PIP against lipid metabolism disorders remains unknown. To test this hypothesis, they examined the intervention of PIP on oleic acid (OA)-induced circadian rhythm disturbance and lipid accumulation in human hepatoma HepG2 cells. The role of the circadian clock genes Bmal1 and Clock in the regulation of PIP lipid accumulation was also investigated. They found that PIP treatment improved lipid metabolism disturbance, redox state imbalance, mitochondrial dysfunction, and OA-induced circadian dysregulation. Meanwhile, the silencing of Bmal1 and Clock genes inhibited the recovery effect of PIP on lipid metabolism disorder in HepG2 cells. The underlying mechanism may be that Bmal1 and Clock genes are involved in the regulation of lipid metabolism-related factors by PIP at protein and mRNA levels. Furthermore, PIP attenuated lipid disturbances by enhancing SREBP-1c/PPARγ and AMPK/AKT-mTOR signaling pathways in a Bmal1/clock-dependent manner. The authors conclude that PIP can indeed prevent and treat obesity-related metabolic disorders by regulating circadian clock genes.

Bay et al. [2] hypothesized that LAT-1-targeted CNS drug-delivery development is important for the treatment of central nervous system and tumors, and established an ultra-performance liquid chromatography-tandem mass spectrometry (UPLC-MS/MS) method for the detection of stable isotope-labeled leucine ([13C6, 15N]-L-leucine), which can be used for functional testing of LAT-1 activity when combined with specific inhibitors, thereby testing the LAT-1 inhibitory ability of new compounds. The authors compared the commercially available human brain capillary endothelial cell line hCMEC/D3 with the in-house developed human brain capillary endothelial cell line NKIM-6, it was found that hCMEC/D3 cells exhibited significantly higher SLC7A5 mRNA expression and the amount of CD98 on the cell surface as well as a high abundance of functional LAT-1HD. In addition, the authors found that JPH203 can achieve a 77% inhibition rate for hCMEC/D3 cells, but only less than half of the inhibition rate for -L-leucine uptake on NKIM-6 cells. The authors conclude that hCMEC/D3 cells are suited for investigations of LAT-1HD-mediated transport over the BBB and LAT-1HD inhibitor screening experiments.

In their study by Chen et al., [3] hypothesized that *Lycium barbarum* polysaccharide (LBP) and capsaicin (CAP) may have a positive effect on the symptoms of ulcerative colitis (UC). Using dextran sulfate sodium (DSS) to induce colitis, they investigate the effect of LBP and/or CAP on colitis symptoms, and determine whether the combination of LBP and CAP can produce further synergistic effects on oxidative stress, inflammatory cytokines, and expression of pain signaling proteins. Their data showed that oral administration of CAP or LBP alone could simultaneously improve abnormal marker concentrations induced by DSS, such as increased serum catalase activity, decreased the serum of interleukin (IL)-6, as well as reduced the protein expression of cyclooxygenase-2 (COX-2), transient receptor potential cation channel V1 (TRPV1) and transient receptor potential ankyrin 1 (TRPA1). However, the synergistic effect of the mixture was only shown in the reduction of serum IL-6 and colonic tumor necrosis factor-α (TNF-α) induced by DSS, and the reduction of COX-2 and TRPA1 protein expression, while the mixture did not improve the effect on indicators of oxidative stress. The authors conclude that the administration of LBP and/or CAP attenuates DSS-induced UC symptoms by inhibiting oxidative stress, proinflammatory cytokines, and protein expression of TRPV1 and TRPA1.

Hou et al. [4] hypothesized that skeletal muscle damage due to sepsis with systemic inflammation caused by severe infection can be improved after administration of L-glutamine (Gln) and/or L-leucine (Leu). Their results indicated that both Gln and Leu are able to suppress monocyte infiltration into muscles, attenuate inflammatory mediator production, inhibit calpain activity, and downregulate HIF-1α mRNA expression in the early and/or late phases of sepsis. Gln had a greater capacity to promote an anti-inflammatory response by polarizing M1 macrophages toward the M2 subtype, while the beneficial effects of Leu treatment focused on suppressing muscle protein breakdown and enhancing the expression of mitochondrial function-related genes. However, Gln administration failed to show synergism with Leu against sepsis-induced muscle injuries. They concluded that Gln promotes the transformation of leukocytes to an anti-inflammatory phenotype to achieve anti-inflammatory effects, while Leu focuses more on enhancing the expression of genes related to mitochondrial function to maintain muscle bioenergetic work. Both Gln and Leu provide beneficial effects in attenuating protein degradation in septic muscle.

In the last study, Chen et al [5] hypothesized that hydroxygenkwanin (HGK) has an inhibitory effect on DNA damage response (DDR)-induced damage and tested it in yeast cells. In addition, the author used liver cancer cells to detect related indicators such as apoptosis and homologous recombination repair and developed a mouse model to confirm the results of in vitro experiments. Using the single-strand annealing (SSA) yeast-cell assay system, they found that HGK can inhibit DDR leading to apoptosis of cells whose DNA cannot be repaired. Furthermore, in vitro experiments showed that HGK enhanced the efficacy of doxorubicin in HCC by inhibiting RAD51-mediated DDR, and in vivo experiments confirmed that HGK enhanced the sensitivity of HCC cells to doxorubicin without any physiological toxicity. They concluded that HGK can inhibit DDR in liver cancer cells and mouse models, making it suitable for use as a chemotherapy adjuvant.

This Special Issue currently brings together a wide range of research, including studies on sepsis-induced muscle injury, brain capillary endothelial cells, inflammatory bowel disease, and metabolic regulation and chemotherapy sensitivity in liver cancer. As the research shows, these diseases have an urgent impact on human health and quality of life. Fortunately, these diseases can obviously be improved and even enhanced drug sensitivity with the treatment of natural active compounds. Therefore, it is hoped that these useful findings will bring new guidelines for the treatment of human diseases.
